# Commitment to Myogenic Differentiation Significantly Aggravates the RNA Phenotype in Myotonic Dystrophy Type 1

**DOI:** 10.1111/nan.70069

**Published:** 2026-03-18

**Authors:** Lise Ripken, Walther J. A. A. van den Broek, Remco T. P. van Cruchten, Jos G. A. Smits, Tabea V. Riepe, Peter A. C. 't Hoen, Derick G. Wansink

**Affiliations:** ^1^ Department of Medical BioSciences Radboud University Medical Center Nijmegen the Netherlands; ^2^ Department of Pathology Radboud University Medical Center Nijmegen the Netherlands; ^3^ Department of Molecular Developmental Biology Radboud Institute for Molecular Life Sciences, Radboud University Nijmegen the Netherlands

**Keywords:** (CTG)n repeat expansion, alternative splicing, differentiation, *DMPK*, MBNL1, myogenesis, RNA sequencing, RNA toxicity

## Abstract

**Aims:**

Myotonic dystrophy type 1 (DM1) is a severe neuromuscular disorder classified as a spliceopathy, caused by a (CTG)n repeat expansion in the 3′ UTR of the *DMPK* gene. The expansion in *DMPK* transcripts sequesters key splicing regulators of the MBNL family, leading to dysregulated alternative splicing. DM1 presents heterogeneous symptoms, with prevalent muscle weakness and myotonia, highlighting the need to understand its impact on the myogenesis process in more detail. This study aims to understand the impact of myotonic dystrophy type 1 (DM1) on myogenesis by investigating RNA expression during the differentiation of DM1 and isogenic CRISPR/Cas9‐corrected DM∆ myoblast cell lines into myotubes.

**Methods:**

RNA samples were collected at various stages of myogenesis from DM1 and control DM∆ myoblast cell lines. Gene expression patterns and alternative splicing signatures were analysed using high‐coverage sequencing.

**Results:**

Proliferating myoblasts exhibited a mild phenotype, with only a few differentially expressed genes and aberrant splicing events. However, upon commitment to fusion in differentiating cultures, there was a marked increase in differentially expressed genes between DM1 and corrected cells, particularly those related to muscle function and ion transport. Notably, aberrant alternative splicing, enriched for MBNL1 binding motifs, aggravated during differentiation, affecting genes associated with muscle organization, contraction and cell junctions.

**Conclusions:**

These findings highlight that the disturbance of myogenesis becomes particularly evident upon commitment to differentiation, emphasising the critical role of differentiation‐ and MBNL1‐dependent splicing throughout myogenesis.

## Summary

- The impact of myotonic dystrophy in skeletal muscle peaks at the proliferation‐to‐differentiation transition in myogenesis.
- Differentiation triggers broad gene‐expression changes, disrupting muscle‐specific pathways.
- MBNL1‐driven splicing defects intensify during differentiation, impairing structural and functional genes.

## Graphical abstract

DM1 myoblasts show mild defects, but RNA toxicity intensifies upon differentiation, where broad gene‐expression changes and escalating MBNL1‐driven splicing defects disrupt muscle‐specific pathways, underscoring a key vulnerability at the transition from myogenic precursor cells to myofibres in patients.

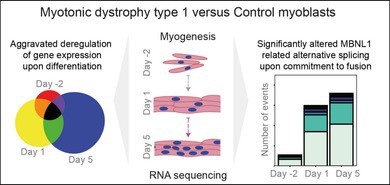

## 1 Introduction

Myotonic dystrophy type 1 (DM1) is the most common adult‐onset neuromuscular disorder with a variety of symptoms, which may include, but are not limited to, skeletal muscle, heart, brain, eyes and the gastrointestinal tract. Muscle‐related problems are very common, with muscle weakness and atrophy being most prominent, particularly in the distal muscles and diaphragm [1]. The underlying mutation is a (CTG/CAG)n repeat expansion in the 3′ UTR of the *DM1 protein kinase* (*DMPK*) and *DM1 antisense* (*DM1‐AS*) gene pair. In DM1, this repeat exceeds the up to 37 triplets observed in healthy individuals due to intergenerational and somatic, tissue‐specific expansion, reaching up to thousands of triplets. The age of onset and disease severity of DM1 are loosely correlated with the number of triplets and are therefore often used for classifying patients into four subtypes [2, 3].

The presence of a (CUG)n expansion in the RNA of *DMPK* is commonly acknowledged as the main pathological mechanism due to its gain‐of‐function effect. The repeat expansion forms complex hairpin structures, sequestering important RNA‐binding proteins (RBPs) *MBNL1* and hyperactivating *CELF1* via the PKC pathway [4, 5, 6, 7, 8]. Both proteins are essential regulators of alternative splicing, RNA localization and stability, and together orchestrate the transition from a foetal to adult transcriptome. Muscle tissue development, regeneration and maintenance are heavily dependent on this meticulously controlled balance of variants [9].

Transcriptomic analysis of DM1 muscle biopsies has shed light on the extensive dysregulation of alternative splicing towards foetal variants in skeletal and heart muscle, where the severity of missplicing could be correlated with inferred MBNL1 concentrations [10, 11]. So far, a multitude of distinct DM1 symptoms have been directly linked to alternative splicing defects, such as *BIN1* and *CLCN1*, to muscle weakness and myotonia, respectively [12, 13]. Additional processes, such as deregulation of PI3K/AKT and AMPK signalling [14, 15], or cellular senescence in the muscle stem cell pool [16, 17], are implied to interfere with muscle cell functioning and/or myogenesis. However, it is still poorly understood at which developmental stage and to what extent the differentiation of muscle progenitor cells into mature myofibres is hampered in DM1.

Previously, we demonstrated in an in vitro set‐up that immortalised myoblasts containing a (CTG)2900 triplet repeat expansion exhibited a clear impairment in myotube formation [18, 19, 20]. This was displayed by a decreased myogenic fusion index (MFI), shorter and thinner myotubes and a lower number of included nuclei per myotube compared with its derivative from which the repeat expansion was excised (DM∆) [18]. A comparative analysis of MBNL1 protein expression revealed a clear imbalance in splice variant usage in DM1, which is already present in proliferating myoblasts and persists throughout the differentiation process towards myotubes. Additionally, total nuclear MBNL1 protein concentrations were significantly lower, likely causing the aberrant alternative splicing of key DM1 cassette exons [21, 22]. All these observations indicate a profound effect of the expanded (CTG)n repeat on myotube formation and muscle differentiation in DM1. However, we still lack a detailed molecular overview of early myogenic processes.

To deepen our understanding of early muscle differentiation impairment in DM1, we have performed an extensive transcriptomic analysis using a cell model comprising DM1 and isogenic CRISPR/Cas9‐corrected (DM∆) myoblasts. We assessed gene expression and alternative splicing at key time points of differentiation, namely, during myoblast proliferation, commitment to fusion and in differentiating myotubes, to provide a comprehensive view of early myogenesis in a well‐controlled in vitro setting. Although myoblasts already displayed a distinctive but mild phenotype with DM1 alternative splicing hallmarks present, gene expression and splice variant usage deviated profoundly from the corrected line upon induction of differentiation. This study provides a more detailed overview of the underlying aberrant mechanisms of myogenesis in DM1.

## 2 Materials and Methods

### 2.1 Cell Culture

Human immortalised myoblasts were cultured in a 1:1 mix of skeletal muscle cell growth medium (Promocell; Heidelberg, Germany) and Ham's F‐10 nutrient mix (Gibco, Carlsbad, CA, USA), supplemented with 20% (v/v) Hyclone Bovine Growth Serum (Cytiva) and GlutaMax (Gibco). Cells were propagated in adherent plastic culture flasks coated with 0.1% gelatin (Sigma‐Aldrich). To maintain proliferation and prevent cell fusion, cell density was kept below 85%. For differentiation, myoblasts were grown to 100% confluency, determined by visual alignment with a brightfield microscope. Cell cultures were washed with 1× PBS and switched for 5 days to differentiation medium consisting of DMEM (Gibco) supplemented with GlutaMax, 10 μg/mL insulin (Sigma‐Aldrich) and 100 μg/mL apo‐transferrin (Sigma‐Aldrich). Both proliferation and differentiation media were refreshed every 2–3 days. Proliferation and differentiation cultures were both maintained at 7.5% CO2 and 37 °C.

### 2.2 RNA Isolation, cDNA Library Preparation and Sequencing

Myoblasts and myotubes were washed with 1× PBS before RNA isolation, performed using the Aurum Total RNA Mini Kit (Bio‐Rad) following the manufacturer's protocol. This included pulling lysates 12 times through a 27G needle and DNase I treatment on the spin column. RNA quality and yield were assessed using absorbance at 260/280 nm (NanoVUE spectrophotometer, GE Healthcare Life Sciences), and RIN scores were consistently above 9.5 (2100 Bioanalyzer, Agilent). RNA library preparation and sequencing were performed by BGI (Beijing Genomics Institute) using the MGIEasy RNA Directional Library Prep Set. In short, mRNA was poly(A) enriched by oligo dT beads, RNA was fragmented and a strand‐specific cDNA library was prepared using random primers for the first strand and dUTP second‐strand synthesis. Sequencing was performed by BGI on its DNBSEQ platform using DNA nanoballs (DNBs) on the DNBseq‐G400. RNA was sequenced with high coverage, resulting in 100 bp strand‐specific paired‐end reads with a minimum of 80 million reads per sample.

### 2.3 RNA Sequencing Data Analysis

The RNAseq datasets analysed during the current study have been deposited in the European Genome‐phenome Archive (EGA) under study number EGAS50000001152. Access to the data is available according to the terms stated in the DAC policy EGAP50000000630, and data requests can be made via the EGA DAC EGAC50000000592, which is associated with the authors and this study.

Fastq files were processed to remove adaptor sequences and low‐quality base calls using Trimgalore v0.6.6. Mapping to the human primary genome assembly GRCh38, release 100 (hg38.100; [23]) was performed with STAR v2.7.0 using multi‐sample 2‐pass mapping. For the second mapping pass, an updated genome index file including novel splice junctions identified in the first mapping pass was used. PCR duplicates were removed from the BAM files, and read counts were generated using the HTSeq framework with the matching GRCh38, release 100 annotation file [24]. The count table was subsequently loaded in R Studio and further analysed. Lowly expressed genes were filtered out by the EdgeR R package v3.38.4 using the filterByExp() with a minimum cutoff at 35 reads across all samples [25]. Cell counts were then normalised for library size and transformed into counts per million (CPM) or log_2_‐CPM (LogCPM).

Principal component analysis (PCA) was performed on the top 2000 most variable genes in the time series on the centred and scaled data using singular value decomposition. Overrepresentation analysis for the top 400 genes of principal components 1 and 2 was subsequently performed using ClusterProfiler v4.4.4 [26]. To identify differentially expressed genes (DEGs), linear model analysis was performed using the limma R package v3.52.4. Data were transformed using the voom option. Contrasts were set to test for significance using linear modelling between DM1 and DMΔ samples at each time point using treat(). Significance was determined at a Benjamini–Hochberg adjusted *p*‐value < 0.05 and an absolute log_2_‐fold change ≥ 1. Visualization of the number and overlapping genes was accomplished using the online *Biovenn* tool [27].

Splice analysis was executed using rMATS 4.1.1 on the same data, realigned to a newly generated human genome index using the second pass option in STAR. Novel splice junctions covered by at least three reads were included. Statistical analyses were based on junction spanning reads only, with a cutoff at an average coverage of 20 reads across all replicates. Significance was called for all splice events with an FDR < 0.05 and a|ΔPSI| > 0.2. Upstream and downstream exons were assigned by rMATS's standard procedure based on lower and higher chromosomal coordinates, independent of strandedness.

Gene expression trends or |ΔPSI| were visualised using the complexHeatmap package v2.21.1 with *k*‐means clustering with 1000 repetitions to improve reproducibility [28]. Gene expression levels were first transformed into logCPM and normalised into *z*‐scores. General expression profiles were calculated based on average *z*‐scores. GO term analysis of biological processes (BP) was performed for over‐representation of genes within the dataset using ClusterProfiler [26]. A *p*‐value cutoff of 0.05 was applied, followed by removing similar GO terms using simplify [26]. Net‐ and heatplots were generated to visualise a selected group of genes.

Binding site analysis for RBPs was performed using the online tool rMAPS2. This tool used the rMATS output files to analyse binding sites near significantly differentially spliced exons; only skipped exons (SEs) were analysed. The default settings (intron 250, exon 50, sliding window size 50, interval 1) and the preset binding motifs for over a hundred RBPs were used for the search [29, 30].

### 2.4 Use of GenAI

ChatGPT and CoPilot were used to improve the clarity and language quality of author‐written text. All AI‐generated suggestions were carefully reviewed and edited whenever necessary before being incorporated into the manuscript.

## 3 Results

### 3.1 DMPK *Expression Is Reduced in DM1 Cells During Early Steps of Myogenic Differentiation*

**FIGURE 1:**
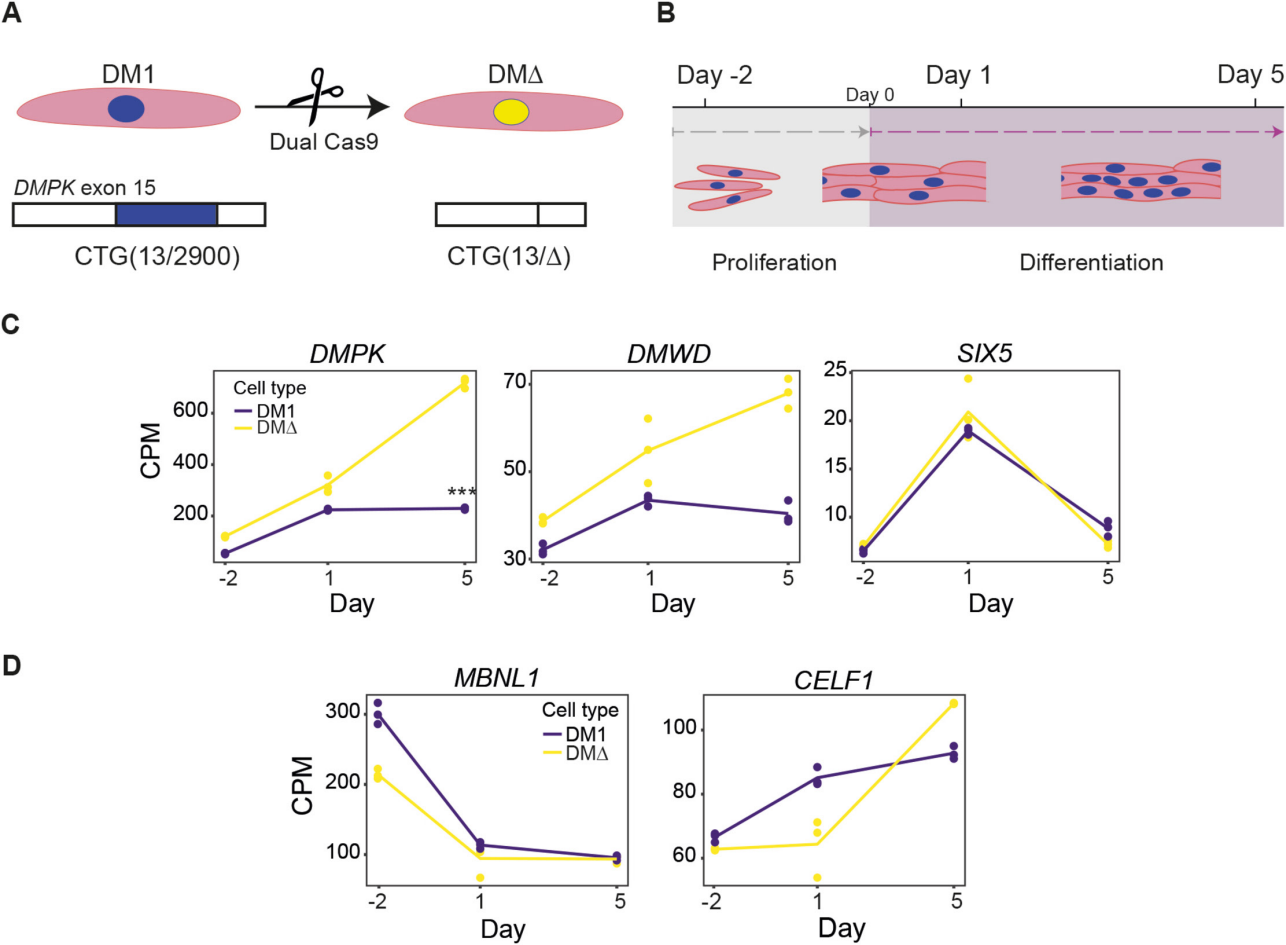
Transcriptome analysis of myoblast to myotube differentiation of DM1 and isogenic, corrected DM∆ cell lines. (A) Congenital DM1 myoblasts (DM1) were previously edited by removing the expanded (CTG)2900 triplet repeat using a dual Cas9 approach to generate the isogenic corrected DM∆ cell line. (B) These myoblasts were grown to 100% confluency, and at Day 0, differentiation was started to induce formation of multinucleated myotubes. RNA samples were taken at Day −2, Day 1 and Day 5. (C) RNA expression of DM1 locus genes represented as counts per million, normalized for library size. *n* = 3 is shown for each cell line. FDR value *** < 0.0001. (D) RNA expression of well‐known RBPs involved in DM1 pathogenesis, represented as counts per million, normalized for library size.

To gain a detailed understanding of transcriptome alterations during aberrant myogenesis in DM1, we performed an in‐depth RNA sequencing study using immortalised myoblasts during differentiation in vitro. The DM1 cell line used in this study harboured at the time of experiments a normal allele with 13 triplets and an expanded allele with around 2900 triplets, determined by optical genome mapping [20]. The well‐characterised isogenic control cell line DM∆, in which the repeat was excised using a dual CRISPR/Cas9 approach, served as a reference for myogenesis in the absence of the expanded repeat (Figure 1A) [19]. To identify RNA expression differences between DM1‐affected and gene‐edited myoblasts, cells were grown to confluency, and differentiation was induced by switching to serum‐free differentiation conditions for 5 days. RNA was isolated for three replicates from cultures at various stages of myogenesis: proliferation (Day −2 before differentiation), commitment to differentiation (Day 1) and myotubes (Day 5) (Figure 1B, Supplemental Figure S1).

Stranded, paired‐end RNA‐sequencing of each sample yielded a minimum of 80 million reads, enabling an in‐depth analysis of both gene expression and alternative splicing, which is known to be severely disturbed in DM1. After quality control and filtering for low read counts, just over 17,000 genes were identified across all timepoints. First, RNA expression levels were retrieved for *DMPK* and its neighbouring DM1 locus genes to study their differentiation‐related regulation of expression and assess potential chromatin‐mediated effects of the repeat expansion. In DM∆ cells, *DMPK* RNA levels consistently increased throughout the three timepoints. However, in DM1 cells, RNA levels plateaued at Day 1 of differentiation, resulting in an approximately three‐fold lower *DMPK* expression in myotubes at Day 5 (Figure 1C). The neighbouring *DMWD* gene, located upstream of *DMPK*, followed the same trend as *DMPK*, but no significant difference was stated here since the fold change was below two. *SIX5* expression was similar in both cell lines throughout.

*MBNL1*, a splicing factor pivotal in DM1 pathogenesis, displayed a consistent downregulation throughout differentiation with no significant difference in RNA expression levels between DM1 and DM∆ cells (Figure 1D). *CELF1*, encoding the second affected RBP whose phosphorylation state is altered in DM1, also showed no significant difference in RNA level between diseased and corrected samples (Figure 1D). Thus, the removal of the repeat expansion primarily affected expression of *DMPK* itself, with only minimal impact, if any, on DM1 locus genes and two important splicing factors involved in DM1 pathogenesis.

### 3.2 DM1 Myoblasts Follow the Main Muscle Differentiation Trajectory

**FIGURE 2:**
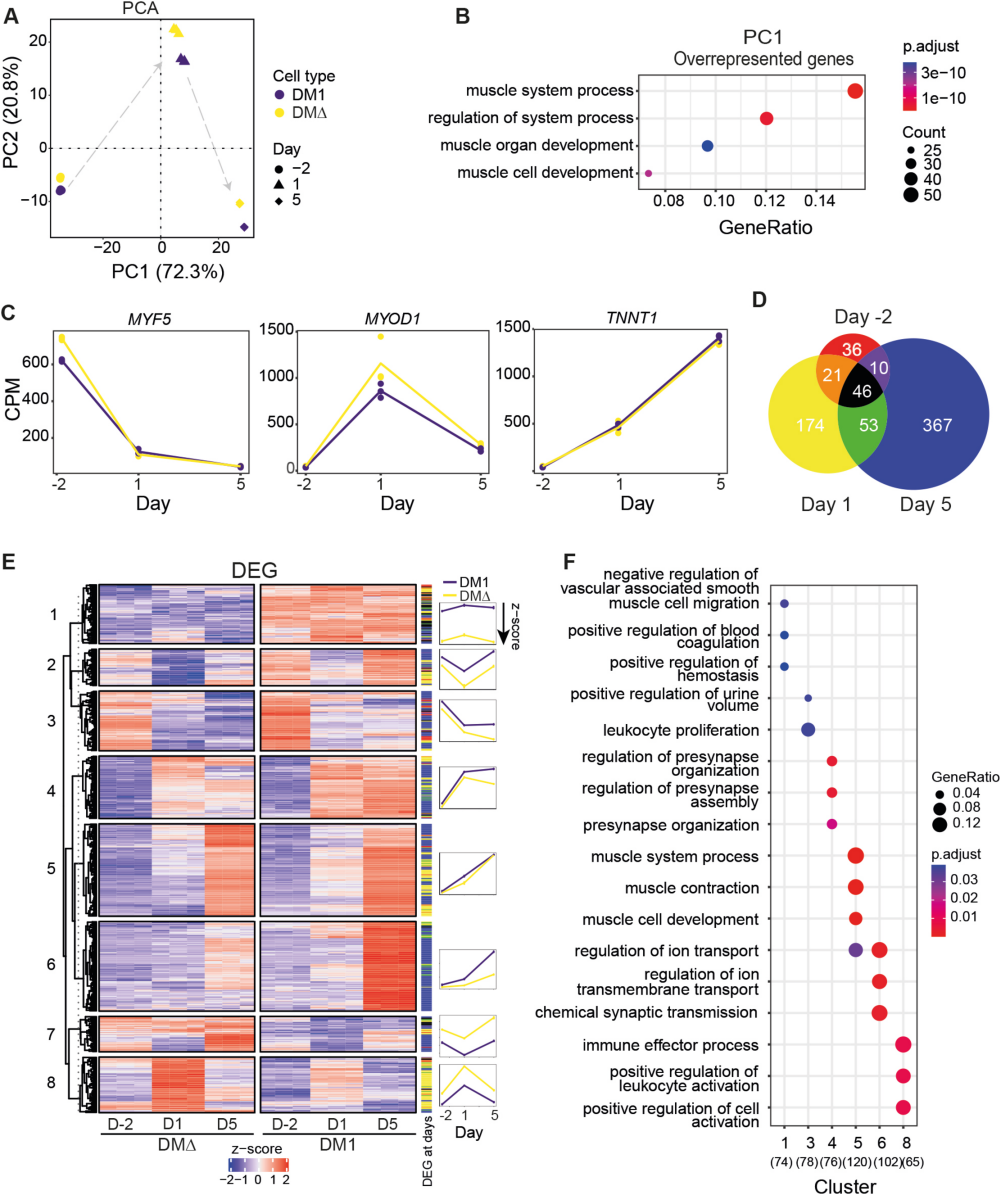
Gene expression analysis of myogenesis in DM1 and DM∆ lines revealed dysregulation of muscle‐relevant gene sets. (A) Principal component analysis was performed on the top 1000 variable genes in the entire dataset. Here, the first two PCs are displayed with percentage variance explained. DM1 and DM∆ cells are shown in blue and yellow, respectively. Arrows indicate time directionality. (B) Overrepresentation analysis of GO biological process terms was conducted on the top 400 loadings of the 2000 genes on PC1. (C) Gene expression (CPM) of three markers of myogenesis. (D) Venn diagram depicting differentially expressed genes at three time points. The area of circles is scaled to the number of contained genes. (E) Heatmap showing *k*‐means (*k* = 8) clustered by Euclidean distance with complete linkage of gene expression, *z*‐score normalized per gene, of all unique DEGs. Colour coding employed in the Venn diagram was used to display at which time point(s) each gene was significantly differently expressed in the column “DEG at day” on the right. The mean *z*‐score was calculated and plotted to demonstrate the overall expression trend during differentiation per cluster. (F) Overrepresentation analysis of GO biological process terms was performed on each separate cluster with the extra simplify command to remove doubles. If cluster is absent, GO terms were not enriched for this group of genes. The number of input genes for GO term analysis is displayed between brackets at the bottom.

PCA was performed to gain an overall insight into the molecular signature of each sample. DM1 and DM∆ cells clustered closely together at each time point, indicating that the most prominent expression patterns were consistent regardless of the presence of the repeat expansion (Figure 2A). Both cell lines exhibited a clear migration pattern across the first two principal components, with principal component 1 primarily influenced by genes associated with the GO terms *muscle system process* and *muscle (organ) development* (Figure 2B). This confirmed both commitment and differentiation towards myotubes for both lines and emphasised that myogenesis was responsible for the largest variation in the dataset.

As expected by the similar behaviour in the PCA analysis, the majority of genes related to the myogenesis hallmark (GSEA) showed no significant alterations between DM1 and DM∆ cells during differentiation (165/190 genes unaltered). Markers of early (*MYF5*), middle (*MYOD1*) and late (*TNNT1*) in vitro myogenesis, for instance, showed similar expression patterns across both cell lines (Figure 2C) [31]. From this, we conclude that DM1 myoblasts demonstrate the capacity to follow early steps of differentiation in a similar manner as the corrected DM∆ cell line, despite significant differences in in vitro myogenic hallmarks, as demonstrated earlier [18, 19].

### 3.3 Myogenic Differentiation Provokes Deregulation of Gene Expression Related to Muscle Processes in DM1

Since others and we have observed profound phenotypic differentiation impairment of DM1 myotubes, we explored which genes and gene expression patterns were affected in DM1 throughout in vitro myogenesis [18, 21, 32]. Pairwise comparisons for DM1 versus DM∆ were performed at each time point using limma voom with biological significance defined as a minimum absolute fold‐change of two and an adj. *p* value < 0.05. Interestingly, the number of DEGs progressively increased as differentiation progressed (Supplemental Data Table 1). While only 113 genes (84 upregulated/29 downregulated in DM1) were differentially expressed at Day −2, this number increased to 294 genes (198 up/96 down) by Day 1 and further rose to 476 genes (374 up/102 down) by Day 5 (Figure 2D). The majority of DEGs at Day 1 and Day 5 were unique, suggesting specific temporal dysregulation. Only 46 genes were consistently deregulated throughout myogenesis, with noteworthy *SOCS2* upregulation in DM1 [33], a gene known to repress skeletal muscle differentiation and involved in insulin signalling. Also, extracellular matrix‐related genes, like *elastin* (*ELN*) and regulatory *AEBP1*, involved in collagen fibril organization, were upregulated in DM1 cells across all time points.

To uncover aberrant global expression patterns throughout the differentiation process, gene expression levels were grouped by *k*‐means clustering and visualised in a heatmap (Figure 2E; Supplemental Data Table 2). Subsequent GO term analysis per gene cluster uncovered a diverse range of affected biological processes, with Clusters 5, 6 and 8 appearing more biologically relevant since a higher ratio of genes within each cluster was represented in a GO term (Figure 2F). Clusters 5 and 6 generally included genes whose RNA levels were elevated in the myotube stage (Day 5). DEGs in these clusters were mostly deregulated on Day 1 and/or Day 5 and were associated with *muscle cell contraction* and *muscle cell development*. Cluster 6 harboured mostly genes that were overexpressed in DM1 at the myotube stage and were related to the *regulation of ion transport* and *chemical synaptic transmission*. Although the other clusters of genes did not reveal a clear biological signature, the presence of these additional deregulated genes may impact cell functioning profoundly in a way not yet understood. Collectively, these findings strongly suggest that the repeat expansion leads to a general deregulation of RNA expression, both in terms of number and functionality, particularly initiating at the onset of differentiation.

**FIGURE 3:**
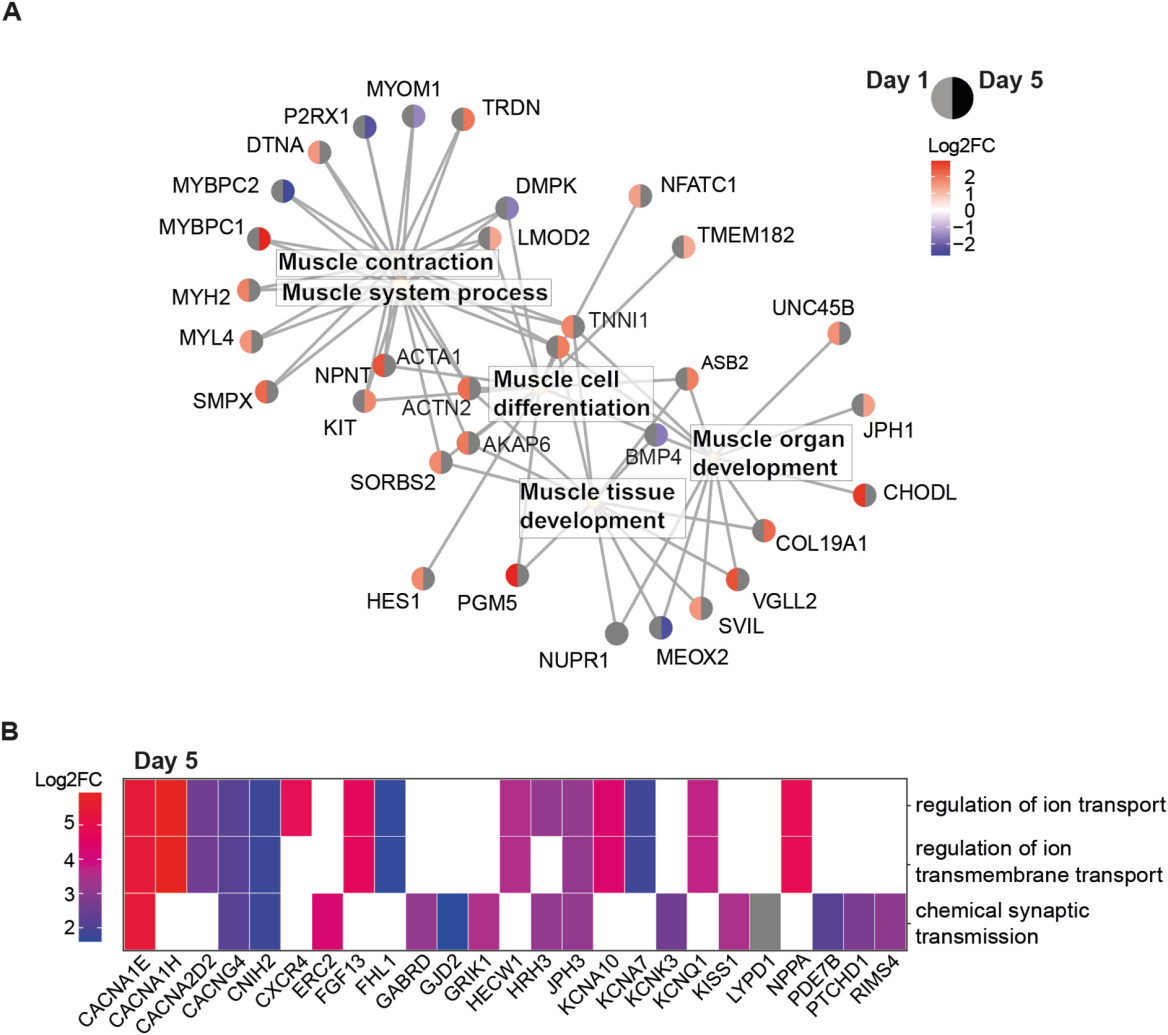
Zoom‐in of aberrant gene expression related to muscle differentiation and ion transport in DM1 cells. (A) GO terms of Cluster 5 of the heatmap (Figure 2) are plotted with related genes in a netplot. Log_2_ fold changes (DM1 vs. DM∆) are shown in colour for DEGs at Day 1 (left half of the dot) and Day 5 (right half). Non‐significant genes are depicted in grey. (B) Log2FC of genes related to GO terms associated with Cluster 6 of the heatmap.

Zooming in on the above‐found biological processes affected during differentiation, we identified interesting candidate genes responsible for an altered myogenesis in DM1 muscles. Within the muscle system processes, an opposing gene expression pattern was observed for *MYBPC1* and *MYBPC2* in DM1 myotubes compared with the rescued cell line at Day 5 of differentiation (Figure 3A). These members of the myosin‐binding protein C family are found in slow and fast striated muscle, respectively. In DM1 myotube cultures, increased levels of RNA for the slow form and decreased levels of RNA for the fast form were found [34, 35]. These proteins have been a recent interest of study again, since their function in muscle organization appears to be vast and not limited only to their structural importance [35]. Interestingly, FHL1, the proposed binding partner of MYBPC protein, is a regulatory gene for NFATC1. Both of these genes were upregulated in our differentiation model, either at Day 5 for *FHL1* (Figure 3B) and Day −2/Day 1 for *NFATC1* (Figure 3A). *NFATC1* is important for fibre type regulation and, together with *FHL1*, is under control of internal calcium levels, known to be deregulated in DM1 [36]. Together with the exclusive upregulation of genes related to *(regulation of) ion (transmembrane) transport*, with the noteworthy five‐Log2 fold higher expression of *CACNA1E* and *CACNA1H* in DM1 compared with DM∆, this further indicates the emergence of a significant dysregulation of ion homeostasis in myotubes, essential for muscle contraction (Figure 3B). In summary, initiating the myogenic differentiation program and committing to fusion appear to trigger cell fate‐dependent misexpression of muscle‐relevant biological processes in DM1 cells.

### 3.4 Characteristic Missplicing in DM1 Exacerbates During Myogenesis

The sequestration of MBNL1 protein by expanded (CUG)n RNA is well‐known to cause widespread alterations in the transcriptome, particularly at the splicing level, both in DM1 patients as well as in cell and mouse models [37]. Our current finding that DM1 myoblasts follow essentially the same differentiation route as corrected myoblasts, based on PCA of gene expression levels, despite impaired myotube formation, prompted us to search the transcriptome for further explanations [18, 21].

**FIGURE 4:**
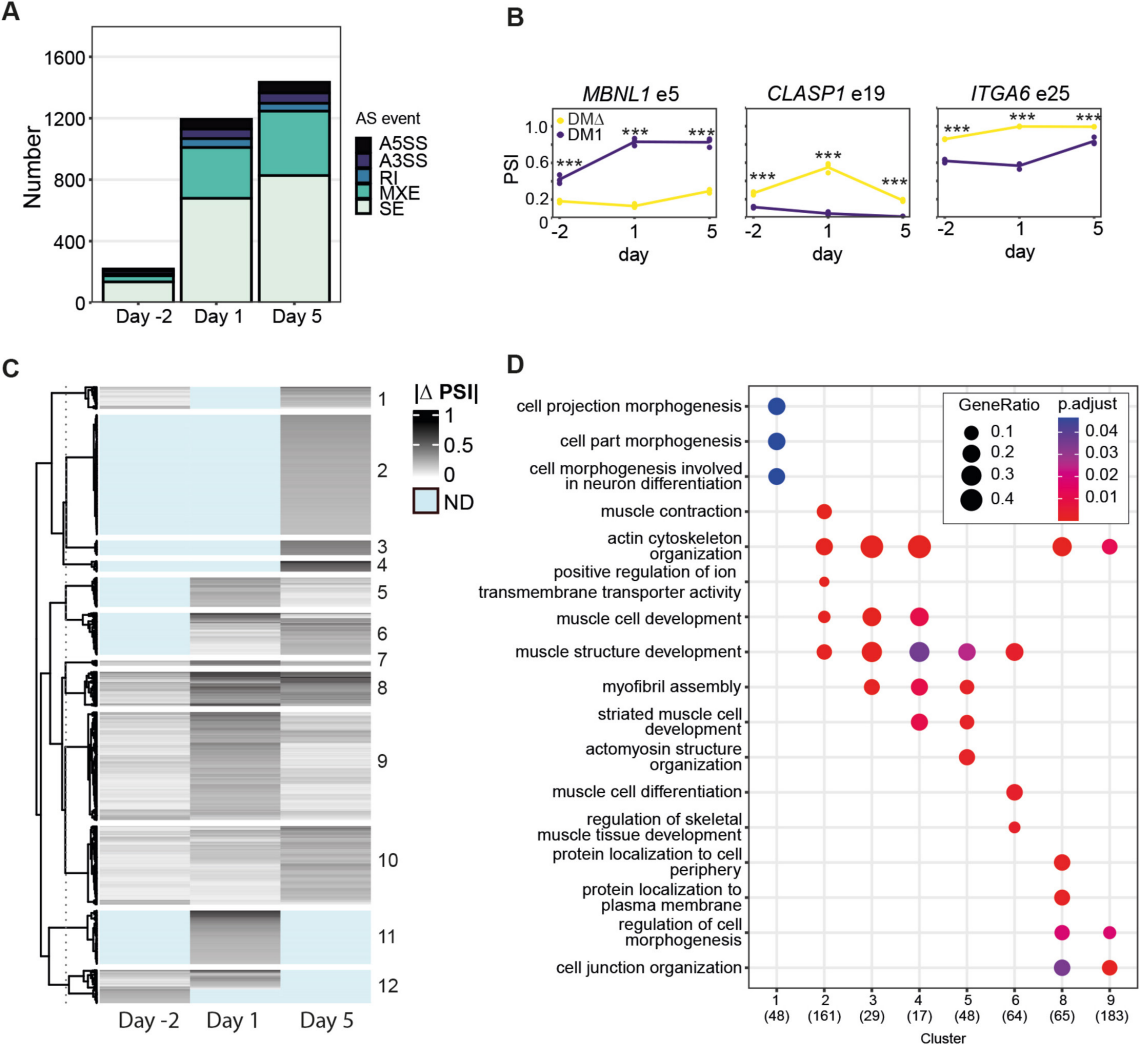
Aberrant alternative splicing increases in DM1 cells upon myoblast commitment to fusion. (A) Absolute number of significantly altered splicing events between DM1 and corrected cells per time point. FDR < 0.05 and|ΔPSI| > 0.2. (B) PSI levels for three SE events. Significance was tested between DM1 and DMΔ for each time point. FDR * < 0.05 ** < 0.01 *** < 0.001. (C) *k*‐means clustered|ΔPSI|levels for all SE events significant on at least one time point. ND is shown for the time points at which a splicing event could not be detected due to low expression. Genes per separate cluster were used as input for (D) to perform overrepresentation analysis of GO biological process terms. Clusters absent from the graph did not contain any enriched GO terms. The number of input genes for GO term analysis is displayed in between brackets at the bottom.

We analysed known and novel alternative splicing events in five categories: alternative 5′ and 3′ splice sites (A5SS/A3SS), retained introns (RI), mutually exclusive exons (MXEs) and skipped exons (SEs), also called cassette exons. Significantly differentially spliced events were identified when the absolute difference between the percentage spliced in (PSI) for DM1 and DM∆ cells was greater than 0.2 (FDR < 0.05 and |ΔPSI| > 0.2). By comparing the absolute numbers of all significantly differentially spliced events, we observed that, in DM1 cells, aberrant alternative splicing was exacerbated at Day 1 of differentiation and in myotubes (Day 5), compared with proliferating myoblasts (Day −2; Figure 4A). This pattern mirrored the trend seen in RNA expression levels. SE was at all time points the dominant type of dysregulated alternative splicing, but events involving abnormal MXEs and alternative 5′ splice site (A5SS) also displayed at least a five‐fold increase upon differentiation. All missplicing modes were thus increasingly affected during myogenesis.

Here, we focused on the most prevalent splicing event, SE. In our dataset, we found well‐known DM1‐related aberrant splicing events, such as *MBNL1* exon 5 (chr3:152,446,704–152,446,757) and *CLASP1* exon 19 (chr2:121,445,449–121,445,496), as well as less acknowledged events, like *ITGA6* exon 25 (chr2:172,501,772–172,501,899) (Figure 4B; Supplemental Data Table 3). Exclusion of this exon is known to result in the use of an alternative stop codon, thereby changing the function and localization of the α6‐Integrin subunit involved in stemness [38].

Clustering all significantly differentially SE events on at least one time point (FDR < 0.05, |ΔPSI| > 0.2) on ΔPSI revealed a diverse heatmap (Figure 4C; Supplemental Data Table 4). Due to low expression levels, some misspliced events could only be detected at one of the three time points, mostly Day 1 or Day 5, while other events could be detected at all time points, but were most severely misspliced at Day 1, Day 5 or both. The severity of missplicing, denoted by ΔPSI, was usually relatively mild at Day −2, but intensified upon commitment to myotubes. As seen in the examples referred to above, the ΔPSI could be increased due to a change in splicing in DM1 cells, while it remained constant in DMΔ cells (*MBNL1* e5), or a change in DMΔ splicing occurred, not seen in DM1 samples (*CLASP1)*, or even more intricate patterns (Figure 4B). In summary, induction of the differentiation program in the DM1 culture caused a profound and increasingly severe imbalance in alternative splicing regulation.

To assess functional overlap within and between splicing events in the heatmap clusters, GO term analysis was performed on the misspliced genes (Figure 4D). Clusters 2 to 6, 8 and 9 all contained splicing events in genes associated with skeletal muscle development in some sort. *Muscle structure development* included genes such as *DMD*, *TTN*, *TNNT2* and *PDLIM3*, while *actin cytoskeleton organization* was associated with these same genes, and also with, e.g., *PXN*, *CLASP1* and *ACTN4* missplicing. With these insights, we concluded that not only the number and degree of missplicing aggravated upon fusion of myoblasts, but it also affected cell fate‐specific genes required for muscle functioning. It must be noted, however, that the majority of misspliced genes were not represented in the GO term analysis. Aside from muscle‐specific aberrations, a general dysregulation of the transcriptome was seen upon the induction of myogenic differentiation, rather than only a single or a limited set of cellular processes going awry.

### 3.5 Enrichment of MBNL1 and RBFOX1 Binding Motifs Flanking Alternatively Spliced Exons in DM1

**FIGURE 5:**
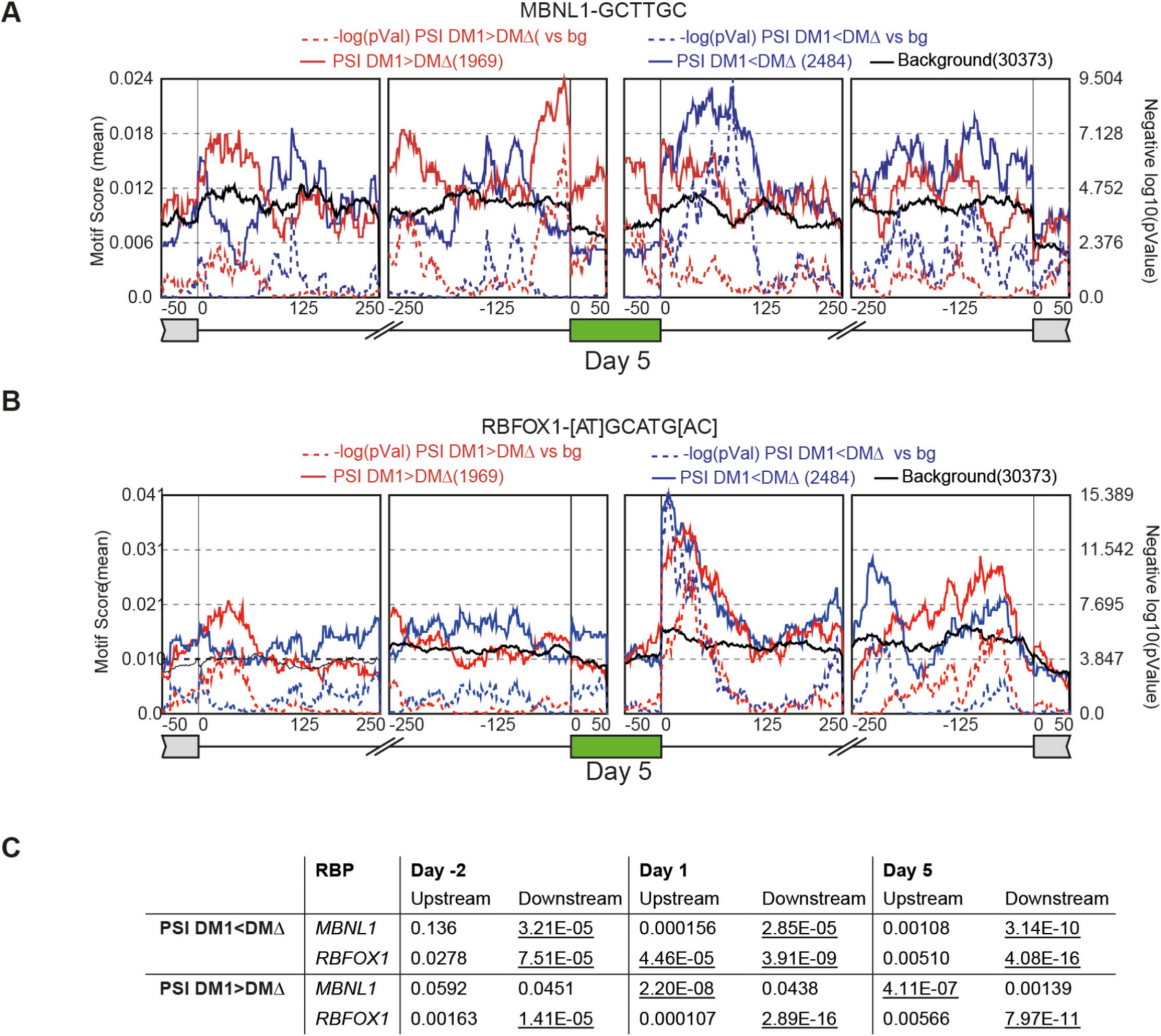
MBNL1 and RBFOX1 motifs are enriched at significantly altered splicing events. Motif enrichment analysis using RMAPS for (A) MBNL1 and (B) RBFOX1 at the alternative spliced exon (green), intronic regions and the upstream and downstream located exons at Day 5 of differentiation. The red and blue lines indicate the presence of the RBP binding motif (motif score) for significantly differentially spliced exons in DM1 compared with DM∆ cell lines. Dashed lines depict the significance (*p*‐value) of the motif scores compared with the motif score of exons that were not differentially used between DM1 and DM∆ (background; black line). (C) The highest *p*‐values of motif enrichment analysis for the intronic region 250 bp upstream and downstream of the target exon at each time point for differentially spliced SE. *p*‐values calculated by a Wilcoxon's rank sum test were interpreted as significant and therefore underlined, with *p*‐value < 0.0001.

Motif enrichment analysis of RBPs was subsequently performed with RMAPS to study which RBPs are most likely involved in the deregulation of the DM1 transcriptome. Shown exemplary for Day 5, but true for all time points, MBNL1 motifs were enriched at intronic regions 250 bp upstream and/or downstream of the alternative exon (Figure 5A,C). MBNL1 motifs downstream of the alternative exon were particularly enriched when PSI values in DM1 myotubes were lower than those in DMΔ. Downstream binding of MBNL1 is known to promote exon inclusion [39, 40]. Reduced MBNL1 availability due to sequestration in DM1, resulting in lower PSI for the exon. In contrast, upstream MBNL1 binding motifs (statistically significant from Day 1 onwards) typically induce exon exclusion. However, in DM1 samples, increased exon inclusion was observed, suggesting a disruption of normal splicing regulation.

Aside from MBNL1, numerous other RBP motifs were enriched at misspliced exons, including those for Quaking (QKI) and PCBP2 (data not shown). Splicing events for muscle (re‐)generation are indeed known to be tightly regulated by multiple RBPs [9, 41]. RBFOX1, suspected of having a competing role with MBNL1, showed motif enrichment predominantly in the downstream intronic region of exons exhibiting both increased inclusion and exclusion (Figure 5B,C) [42]. This suggests that, while RBFOX1 binding may be associated with aberrant splicing, it does not confer a consistent directionality of effect, unlike MBNL1. Other factors, such as alternative, possibly more effective, RBPs (including MBNL1 itself), or gradients of RBPs, could play a role here [43]. All in all, our analysis supports the notion that aberrant alternative splicing in DM1 is primarily moderated by MBNL1, with a potential modulatory role for RBFOX1, and showcases the increased reliance on precise splicing regulation during myogenesis.

## 4 Discussion

Patients with DM1 suffer greatly from muscle‐related symptoms, and several studies have revealed a relation between disease severity and aberrant alternative splicing [44, 45]. However, it is still unclear how the early stages of muscle development are deregulated in the disease, starting at the stem cell level. To gain insight into the underlying mechanism, we employed an in vitro muscle cell model—ideally suited for studying early myogenic events—and conducted an extensive transcriptomic analysis at three key time points. At each stage, the DM1 cell line carrying a (CTG)2900 repeat was compared with its isogenic, repeat‐excised derivative DM∆. Importantly, DM∆ myoblasts exhibited a markedly enhanced myogenic differentiation capacity in vitro—e.g., improved myogenic fusion index, larger myotubes and corrected missplicing, as previously reported by our group [18]. This comparative set‐up provided a clear and broad overview of the earliest myogenic processes affected in DM1, with the most notable being the aggravated deregulation of gene expression and alternative splicing once myoblasts commit to myotube differentiation.

DM1‐burdened muscle cells did not appear to deviate greatly from their repeat‐excised counterparts based on PCA. We found that DM1 and DM∆ cells followed a similar differentiation trajectory, indicating that DM1 myoblasts were able to exit the cell cycle and continue into myogenesis. However, mRNA expression patterns, such as those of slow and fast *MYBPC*s and *NFATC1*, were significantly altered when exiting the proliferative myoblast phase. Previous studies on these myotubes already revealed a thinner and shorter morphology, indicative of impaired fusion capabilities [18]. While DM1 patients usually exhibit relatively normal muscle development, congenital DM1 (cDM) patients display increased satellite cell numbers at birth, delayed muscle maturation and a higher prevalence of type 1 muscle fibres [46, 47, 48]. Thus, progression into myogenesis, specifically the crucial switch from proliferation into the differentiation phase, particularly reveals the effect of the toxic (CTG)n repeat expansion in muscle. Notably, our in vitro study was confined to the first steps of myogenesis to avoid selection bias due to snapping of myotubes upon contractile behaviour [49]. Additionally, p16‐mediated premature senescence was likely masked in our cells, since myoblast immortalization was performed by lentiviral expression of human telomerase and overexpression of the p16 ligand, CDK4 [16, 32, 50].

Zooming in on the absolute numbers of deregulated genes, cells later in myogenesis were more heavily affected by the presence of the (CTG)n repeat expansion than proliferating myoblasts. This observation indicates that, in DM1, myoblasts exhibit distinct but minimal alterations, while molecular processes in myotubes are significantly impaired. It has been previously reported that myotubes are differently impacted by DM1 compared with myoblasts, although the severity of this varies [51, 52]. These varying outcomes could be attributed to the use of cell types with a diverse genetic background as compared with our well‐characterised isogenic cell lines. The stark difference between DEG numbers in myoblasts and myotubes could potentially be explained by the target genes of downstream actors of expanded *DMPK* RNA toxicity, namely, the MBNL and CELF1 proteins. Most genes deregulated in the myotube phase were either minimally expressed or not expressed at all in myoblasts. Our findings stress that DM1 disease load is not equally distributed throughout the myogenic pathway, also emphasising the importance of choosing the right cell model to study therapeutic strategies.

In cells harbouring long repeat expansions, hypermethylation of CpG sites near the repeat might cause local chromatin remodelling, leading to lower *DMPK* levels, an effect that has recently been described to be enlarged by a more mature differentiation status [52]. This mechanism may also affect genes further up and downstream, *DMWD* and *SIX5*, possibly attributing to a DM1 phenotype observed in transgenic mice [53]. In our study here, using the DM∆ line, we have not found any statistically significant alteration in neighbouring *DMWD* and *SIX5* expression, likely since hypermethylation upstream of the repeat expansion was irreversibly present still after CRISPR/Cas9‐mediated excision of the repeat [54]. Interestingly, *DMPK* levels failed to increase in DM1 upon initiation of differentiation, as previously reported [18]. This suggests a regulatory mechanism that operates independently of methylation status. It seems improbable that the observed decrease in *DMPK* levels reflects challenges in isolating expanded *DMPK* mRNA, given that the RNA isolation protocol was uniformly applied at all time points.

DM1 myotubes showed upregulation of slow (*MYBPC1*) and downregulation of fast (*MYBPC2*) myosin binding protein transcripts compared with gene‐edited myotubes, consistent with RNA sequencing findings in both *HSA* ^LR^ as well as in *Mbnl1* KO mice, suggesting an MBNL1‐dependent process, potentially via alternative splicing [55]. A recent DM1 mouse study has shown that, as a consequence of *Clcn1* missplicing, a higher number of oxidative (slow) fibres were observed in DM1 compared with WT muscle [56]. ASO treatment promoting *Clcn1* exon 7a skipping in this DM1 model resulted in a specific reduction of type 2A oxidative muscle fibres. In our in vitro model, we observed a trend towards a more oxidative fibre type in DM1, indicated by the upregulation of type 2A *MYH2* at Day 1, *MYL2*—typically associated with type 1 fibres—at Day 5 and ACTN2 at Day 1 and Day 5, although the latter was not statistically significant on Day 5 [57]. The 2D differentiation environment allowed only reliable conditions for differentiation up to 5 days, which clearly limits maturation of the myotubes and expression of adult MHC forms. The observed imbalance between slow and fast MYBPCs in our study may already be a forerunner of a more oxidative phenotype in the myotubes, making it a focus for future in vitro studies.

The onset of an altered muscle trajectory is also evident in the pronounced calcium‐dependent deregulation, marked by the upregulation of *NFATC1*, *FHL1* and various ion channels. Altered splicing of *ATP2A1* (or SERCA) in DM1 leads to increased intracellular calcium levels, activating successively calmodulin, calcium/CaM‐dependent kinases (CaMK) and phosphatases [36]. Calcineurin, one of the phosphatases, stimulates the activity of NFATC1 via subunit A (CnA), enabling the expression of the slow oxidative program in muscle, such as the upregulation of MyHC IIA. In our study, increased expression of *NFATC1* was apparent from Day −2, expression increased notably during myogenesis, and altered ion homeostasis was apparent at Day 5. Thus, while this in vitro model produced immature myofibres, it successfully allowed for the detection of key myogenic processes that are disrupted in DM1.

Aberrant missplicing was clearly aggravated upon commitment to differentiation. The role of MBNL1, which is irrefutably linked to DM1 pathomechanisms due to its sequestration by (CUG)n‐containing RNA, likely plays a profound role in this temporal dysregulation. Knockout MBNL cell models mimic the dysregulation in alternative splicing in DM1, with common splicing events being, for example, *NFIX* exon 7 and *DMD* exon 78, also misspliced in our study [58]. Interestingly, the KO models also displayed impairment in myogenesis, like impaired fusion capacities in iPSC‐derived MBNL1 KO cell models and skeletal muscle abnormalities, specifically myotonia (6 weeks postnatal), abnormal nuclear positioning and myofibre splitting in *Mbnl1* KO mouse model [58, 59]. Together with our data on the involvement of MBNL1 binding motifs in dysregulated SEs, this clearly implies an MBNL1‐mediated impairment in the transition of myoblasts to myotubes. Whether this is only because of increased *DMPK* levels upon commitment to differentiation leading to lowered functional MBNL1 levels, or supported by a myogenic differentiation‐related change in *MBNL1* expression (i.e., alternative transcription initiation, splicing or polyadenylation), or a greater dependence on processes mediated by MBNL1‐targeted variants, is unclear.

Given the diversity in age‐of‐onset and disease manifestation in DM1 and its correlation to repeat length [3, 60], our findings prompt several considerations for interpreting repeat‐length dependent effects in muscle, especially in light of marked cell‐to‐cell variability [61]. Although (CTG)n‐repeat length is typically measured in blood, it is well established that the repeat is substantially longer in skeletal muscle in patients. As a result, the actual repeat lengths present in satellite‐cell nuclei and individual myofibre nuclei in patients remain largely unknown, despite being most relevant for pathogenic outcome. The (CTG)2900 cell line studied here presumably reflects an early‐onset phenotype similar to that observed in young patients, including those with congenital DM. Extending our analyses to additional, ideally isogenic, cell lines carrying substantially shorter (100–500 CTGs) or much longer (> 5000 CTGs) repeats would be highly informative. Since somatic instability drives repeat expansion over time, comparing a broad range of repeat lengths will provide important insights into temporal and repeat‐length‐dependent disease mechanisms in muscle, including those influencing age‐of‐onset.

A potential challenge in such studies is that toxic effects associated with relatively short repeats (e.g., < 500 CTGs) may not manifest within the limited timeframe of typical experimental windows. In patients who inherit shorter repeats, pathogenic changes may develop slowly as a consequence of cumulative effects and become apparent only after many years, whereas very long repeats may drive more acute processes that are directly measurable in standard in vitro assays. Thus, the (CTG)2900 line may also approximate the situation in cells from older patients, in whom prolonged somatic expansion has produced substantially longer repeats over time.

A possible alternative to using cell lines is the analysis of patient muscle biopsies. However, beyond practical constraints related to obtaining sufficient, relevant and comparable tissue samples, these biopsies contain the full spectrum of cell types present in muscle, not only satellite cells and myofibres. In this respect, the cell lines used in this study provide a much “cleaner” and more controlled model system for studying myogenic mechanisms in DM1 muscle. More importantly, the inherent somatic mosaicism in patient muscle samples produces a range of repeat lengths, making it difficult to assess potential relationships between specific repeat lengths and particular disease mechanisms.

In conclusion, using an isogenic set of DM1 and repeat‐excised derivative cell lines, we show here that the commitment‐to‐fusion phase is affected in myoblasts with a long repeat in DM1, causing mild impairment in progenitor cells but more pronounced effects in mature muscle cells. MBNL1 is an important mediator of this temporal dysregulation by driving missplicing of specific variants. Current therapeutic interventions mainly aim to increase the concentration of free MBNL1, which could potentially reverse the dysregulation, restore muscle regeneration and mitigate the disease phenotype. Comparative studies across (CTG)n‐repeat lengths will be key to understanding differential RNA‐mediated muscle pathomechanisms that depend on differentiation state, repeat length and time.

## Author Contributions

Conceptualisation: **Lise Ripken**, **Walther J.A.A. van den Broek** and **Derick G. Wansink**. investigation: **Lise Ripken** and **Walther J.A.A. van den Broek**. validation: **Lise Ripken** and **Jos G.A. Smits**. formal analysis: **Lise Ripken**, **Remco T.P. van Cruchten** and **Tabea V. Riepe**. writing – original draft: **Lise Ripken** and **Derick G. Wansink**. writing – review and editing: **Lise Ripken**, **Derick G. Wansink** and **Peter A.C.’t Hoen**. visualization: **Lise Ripken**. supervision: **Derick G. Wansink**.

## Funding

This work was supported by Prinses Beatrix Spierfonds (grant numbers W.OR18‐06 and W.OR23‐03) and AFM‐Téléthon (grant number 24975).

## Ethics Statement

This study used established human cell lines and involved no human participants, identifiable data or animal experiments; hence, no ethical approval or informed‐consent procedures were required.

## Conflicts of Interest

The authors declare no conflicts of interest.

## Supporting information


**Figure S1:** (A) Representative images of proliferating and differentiating muscle cell cultures, taken shortly before RNA isolation. Scale bar represents 75 μm.

**Table S1:** Supporting Information.

**Table S2:** Supporting Information.

**Table S3:** Supporting Information.

**Table S4:** Supporting Information.

## Data Availability

The RNAseq datasets analysed during the current study have been deposited in the European Genome‐phenome Archive (EGA) under study number EGAS50000001152. Access to the data is available according to the terms stated in the DAC policy EGAP50000000630, and data requests can be made via the EGA DAC EGAC50000000592, which is associated with the authors and this study.
