# Supplementary material for: Commitment to Myogenic Differentiation Significantly Aggravates the RNA Phenotype in Myotonic Dystrophy Type 1
Source: Neuropathol Appl Neurobiol. 2026 Mar 18;52(2):e70069. doi: 10.1111/nan.70069 (PMC12997521; doi:10.1111/nan.70069)
Supplement: Supplementary file 1 — Figure S1: (A) Representative images of proliferating and differentiating muscle cell cultures, taken shortly before RNA isolation. Scale bar represents 75 μm. [file NAN-52-e70069-s001.pdf]

Supplementary figures

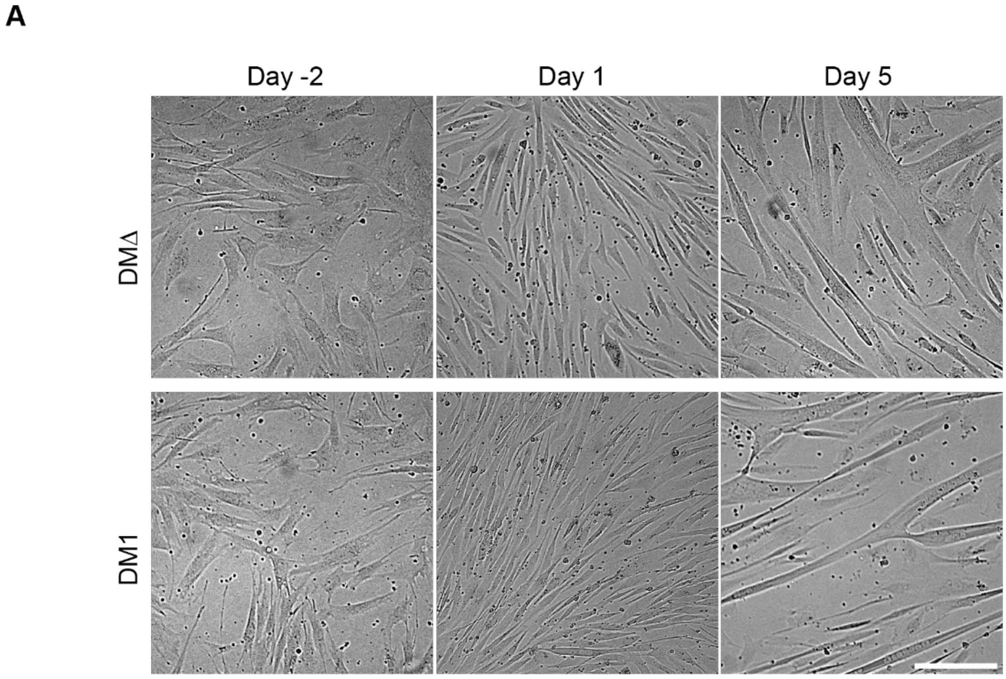

Supplemental Figure S1: (A) Representative images of proliferating and differentiating muscle cell cultures, taken shortly before RNA isolation. Scale bar represents 75 µm.
